# Epidemiology, Etiology, and Treatment of Isolated Cleft Palate

**DOI:** 10.3389/fphys.2016.00067

**Published:** 2016-03-01

**Authors:** Madeleine L. Burg, Yang Chai, Caroline A. Yao, William Magee, Jane C. Figueiredo

**Affiliations:** ^1^Department of Medicine, Keck School of Medicine, University of Southern CaliforniaLos Angeles, CA, USA; ^2^Center for Craniofacial Molecular Biology, Ostrow School of Dentistry, University of Southern CaliforniaLos Angeles, CA, USA; ^3^Division of Plastic and Reconstructive Surgery, Keck School of Medicine, University of Southern CaliforniaLos Angeles, CA, USA; ^4^Division of Plastic and Maxillofacial Surgery, Children's Hospital Los AngelesLos Angeles, CA, USA; ^5^Department of Preventive Medicine, Keck School of Medicine, University of Southern CaliforniaLos Angeles, CA, USA

**Keywords:** cleft palate, genetics, risk factors, etiology, treatment

## Abstract

Isolated cleft palate (CPO) is the rarest form of oral clefting. The incidence of CPO varies substantially by geography from 1.3 to 25.3 per 10,000 live births, with the highest rates in British Columbia, Canada and the lowest rates in Nigeria, Africa. Stratified by ethnicity/race, the highest rates of CPO are observed in non-Hispanic Whites and the lowest in Africans; nevertheless, rates of CPO are consistently higher in females compared to males. Approximately fifty percent of cases born with cleft palate occur as part of a known genetic syndrome or with another malformation (e.g., congenital heart defects) and the other half occur as solitary defects, referred to often as non-syndromic clefts. The etiology of CPO is multifactorial involving genetic and environmental risk factors. Several animal models have yielded insight into the molecular pathways responsible for proper closure of the palate, including the BMP, TGF-β, and SHH signaling pathways. In terms of environmental exposures, only maternal tobacco smoke has been found to be strongly associated with CPO. Some studies have suggested that maternal glucocorticoid exposure may also be important. Clearly, there is a need for larger epidemiologic studies to further investigate both genetic and environmental risk factors and gene-environment interactions. In terms of treatment, there is a need for long-term comprehensive care including surgical, dental and speech pathology. Overall, five main themes emerge as critical in advancing research: (1) monitoring of the occurrence of CPO (capacity building); (2) detailed phenotyping of the severity (biology); (3) understanding of the genetic and environmental risk factors (primary prevention); (4) access to early detection and multidisciplinary treatment (clinical services); and (5) understanding predictors of recurrence and possible interventions among families with a child with CPO (secondary prevention).

## Introduction

Isolated cleft palate (CPO) is the least common form of oral clefting (approximately 33% of all oral clefts), affecting 1 to 25 per 10,000 newborns worldwide. Because of the rarity of CPO and its distinct embryologic origins and recurrence risks from cleft lip with or without cleft palate (CL/P), most studies either exclude CPO or conflate these cases with CL/P due to hypothesized common genetic and epidemiologic risks, although no studies to date have had sufficient power to evaluate these hypotheses. Thus, our knowledge about whether the risk factors for CPO do indeed differ from those of CL/P remains incomplete. Although much remains to be confirmed in human studies, there is a wealth of information from animal models on the molecular biological pathways necessary for complete closure of the primary and secondary palate during embryogenesis. In this article, we summarize the results of epidemiologic population-based studies and animal models to present a comprehensive review of the anatomy and classification, descriptive epidemiology, molecular biology, risk factors, treatment, and outcomes for children born with a cleft palate.

## Anatomy, embryology, and classification

### Anatomy

**Figure 1:**
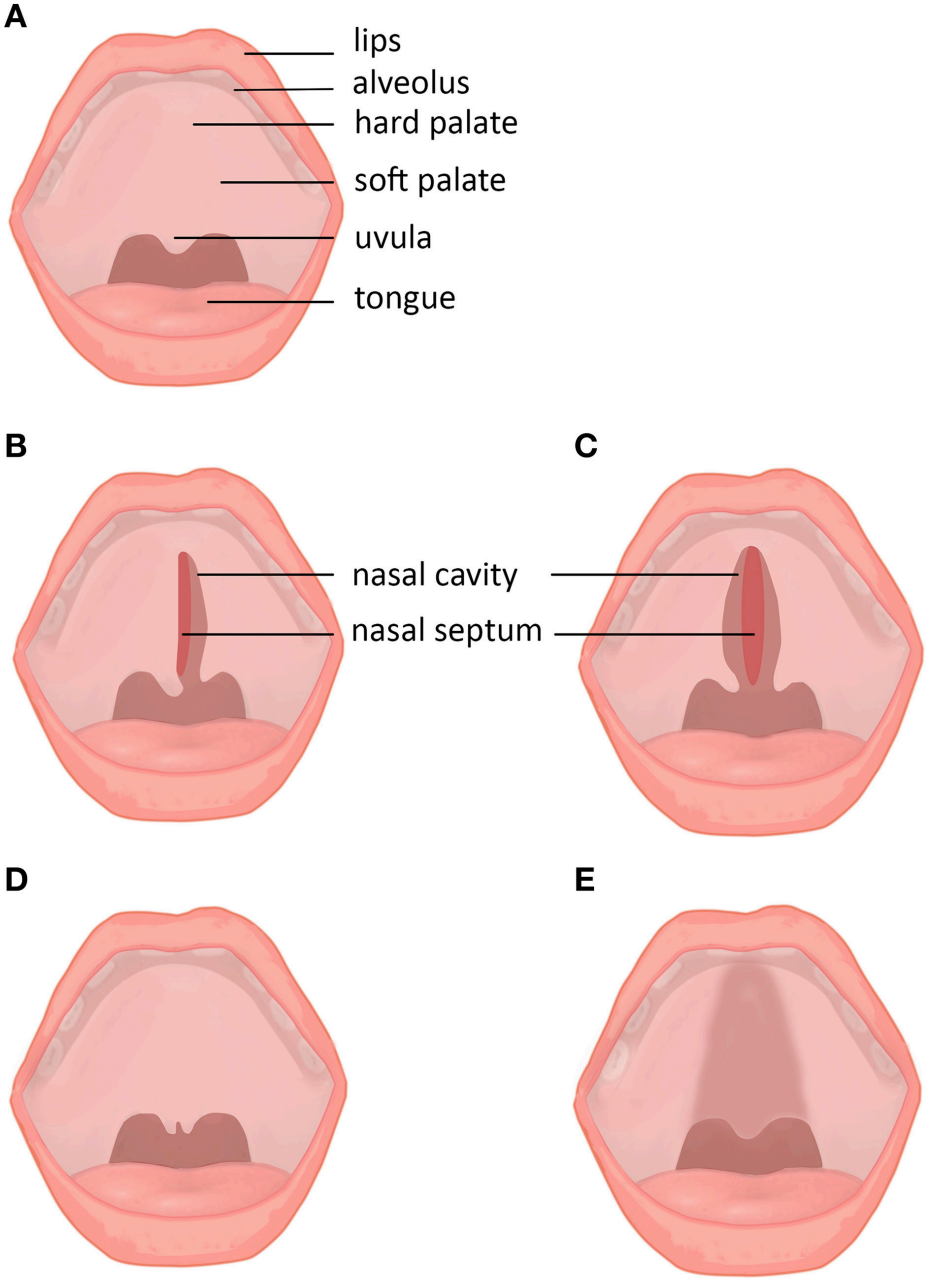
**Subtypes and subclinical forms of cleft palate**. **(A)** Normal lip and palate. **(B)** Unilateral cleft palate. **(C)** Bilateral cleft palate. **(D)** Cleft uvula. **(E)** Submucous cleft palate.

The human palate (Figure 1) consists of a bony *hard palate* and fibromuscular *soft palate*. The hard palate is further divided into *primary* and *secondary* portions. The *primary* palate lies anterior to the incisive foramen, and the *secondary* palate lies posterior separating the nasal passage from the pharynx (Wexler, 1997; Friedman et al., 2010).

The *soft palate*, or velum, is a fibromuscular shelf forming a sling posterior to the hard palate and consisting of five pairs of muscles: the palatoglossus, palatopharyngeus, levator veli palatini, tensor veli palatine, and musculus uvulae. The palatoglossus and palatopharyngeus muscles are superficial on the oral side and help draw the soft palate downward and lateral pharyngeal walls inward. Deep within these muscles are the musculus uvulae, which pull the uvula forward and upwards. The tensor veli palatini tenses and depresses the soft palate while opening the Eustachian tube. The levator veli palatini, the largest muscle in the group, elevates the soft palate and secondarily opens the Eustachian tube (Wexler, 1997).

### Embryology

Embryonic development of the palate occurs between the 4th and 12th to 13th weeks of life. During that time, the basic morphology of the face is formed with the fusion of the five basic facial prominences: the midline frontonasal and the paired maxillary and mandibular prominences (Afshar et al., 2012). The medial portion of the frontonasal prominence gives rise to the primary palate, while the maxillary prominences create the secondary palate. Each facial prominence consists of neural crest cells, which are ectodermal-derived cells at the margins of the neural folds bilaterally and the transitional area between the neuroectoderm and epidermis, in segmental positions along the neural tube (Afshar et al., 2012). Neural crest cell migration into the craniofacial and pharyngeal complexes is predetermined by inductive events between the forebrain, midbrain, and hindbrain, the timing and extent of which is dependent on a complex pattern of gene signaling, including *Hox, Ssh, Otx, Gsc, Dlx, Msx, Lhx*, and *Prrx based on animal studies* (Sperber, 2002a; Chai and Maxson, 2006). Deficiencies in neural crest cell migration or proliferation are the source of a diverse spectrum of craniofacial malformations, including cleft palate (Hall, 1999; Eppley et al., 2005).

The primary palate forms around developing olfactory placodes with rapid proliferation of lateral epithelium and underlying mesenchyme, controlled in part by FGF, BMP, SSH, and retinoic acid (Mossey et al., 2009). Separation of the oral and nasal cavities occurs with fusion of the frontonasal process and maxillary processes; fusion requires coordinated growth between the processes and apoptosis of the epithelium that forms the transient nasal bridge (fin) between the paired processes (Mangold et al., 2011). Clefting of the primary palate most often occurs between the primary and secondary palates at the incisive foramen that separates the lateral incisors and canine teeth; initial mesenchymal deficiency, delayed ossification, decreased premaxilla volume, increased apoptosis, or increased bone resorption due to a lack of functional forces on the primary palate have been identified as sources of clefting (Siegel et al., 1985, 1991; Mooney et al., 1992).

Closure and fusion of the secondary palate requires timed interactions, movements, and apoptosis along the medial margins of the palatal shelves (Zhou et al., 2013). Secondary palate fusion occurs from anterior to posterior, beginning at the incisive foramen and concluding with uvular fusion (Smith et al., 2012). Starting at week 8, the palatal shelves rotate from a vertical position surrounding the tongue and elevate into a horizontal position (Sperber, 2002b). This movement is slightly delayed in females (Burdi and Faist, 1967). After shelf rotation and elevation, adhesive contact, seam fusion along the medial edges, and apoptosis of the epithelium are critical for normal secondary palatogenesis (Sperber, 2002b). As the secondary palate closes, the mandibular prominences grow and the tongue becomes positioned more anteriorly in the oral cavity (Diewert and Lozanoff, 2002). Clefting of the secondary palate may arise from failure of the palatal shelves to elevate, adhere or fuse, which may be due to genetic, mechanical or teratogenic factors that perturb the stepwise growth, rotation, and fusion of the prominences (Afshar et al., 2012). Factors that have been shown to impede palatal shelf contact include delayed shelf rotation into the horizontal position, small palatal shelf size, deficient extracellular matrix accumulation, delayed growth of mandibular prominences, head extension (leading to an increase in the vertical facial dimension), abnormal craniofacial morphology, abnormal first arch development, increased tongue obstruction of shelf movement secondary to mandibular retrognathia, and amniotic sac rupture leading to severely constricted fetal head and body posture (Diewert and Lozanoff, 2002; Johnston and Bronsky, 2002).

Palatal clefts span many degrees of severity and can include the soft palate, hard palate and alveolus (the bony ridge of the maxilla or mandible that supports and contains teeth). The degree of palate clefting is a consequence of the point in fetal development at which formation was disrupted (Friedman et al., 2010). Primary palate fusion is usually complete the during the fourth to 8th week, while the secondary palate starts forming during the 8th week, with completion by about the 12th week (Marazita and Mooney, 2004; van Aalst et al., 2008). This difference in timing between the primary and secondary palate is one reason for considering CL/P and CPO as different developmental deformities. Additionally, the female palate is known to close 1 week later than the male palate, increasing the risk of cleft palate formation, and is a current hypothesis for the higher frequency of cleft palate in females. Burdi and Silvey demonstrated this by finding the critical week of palatal closure to be the 7th week in males and 8th week in females (Burdi and Silvey, 1969; van Aalst et al., 2008).

### Classification

Clefts affecting the palate are grossly classified as unilateral (incomplete vs. complete), bilateral (incomplete vs. complete), or submucous. A number of descriptive classifications have been presented. In 1931, Veau classified clefts into four groups: (1) soft palate cleft only; (2) cleft of soft and hard palate; (3) unilateral cleft lip and palate; (4) bilateral cleft lip and palate. However, this classification does not address primary palate clefts or distinguish incomplete vs. complete clefts of the lip and palate (Veau, 1931). Kriens introduced a palindromic classification using the acronym “LAHSHAL,” describing the bilateral anatomy of the lip (L), alveolus (A), hard (H), and soft (S) palates from right to left. The first character is for the patient's right lip, and the last character is for the patient's left lip. The LAHSHAL code indicates complete cleft with a capital letter and an incomplete cleft with a small letter. For example, a complete right-sided unilateral cleft lip, alveolus, hard and soft palate is “LAHS.” This is the system currently used in the outcomes registry for the American Cleft Palate and Craniofacial Association.

## Non-syndromic vs. syndromic cleft palate

Cleft palates can be divided into two groups: (1) syndromic CPO is associated with additional structural abnormalities occurring outside the region of the cleft (also called “nonisolated” cleft palate) or with a syndrome with a known genetic etiology, and (2) non-syndromic CPO is an isolated condition unassociated with any recognizable anomalies (also known as “isolated” cleft palate; Mai et al., 2014; Watkins et al., 2014). The proportion of oral clefts with additional anomalies is more frequent for CPO than for CL/P (Mossey et al., 2009). About 50% of CPO are associated with another malformation syndrome, compared with less than 15% of CL/P (Shprintzen et al., 1985). The most commonly associated anomalies associated with CPO are congenital heart defects (31.1%), deformations (22.4%), hydrocephaly (11.2%), urinary tract defects (9.7%), and polydactyly (9.2%; Mossey and Catilla, 2003).

Originally, non-syndromic CPO was thought to be a distinct condition with its own genetic etiology, separate from all forms of syndromic CPO, partially due to the low occurrence of non-syndromic and syndromic forms of CPO within the same family. However, recent research has shown both conditions may be opposite ends of a large spectrum of CPO, largely due to advances in genome sequencing and recognition of subclinical phenotypes. For CPO associated with a syndrome with a known genetic cause, many of these syndromes were thought to have a set group of physical features when first discovered. As genome sequencing has become more common in the clinical setting, new cases of these syndromes are now being diagnosed in patients who have the genetic marker but do not always display all of the characteristic features, and may display additional features as well. In non-syndromic CPO, recent recognition of subclinical phenotypes, such as bifid uvula and submucous cleft palate, has led to an increase in the number of affected family members for some patients, shedding light on possible inheritance patterns (Reiter et al., 2012; Watkins et al., 2014). In both non-syndromic and syndromic forms of CPO, the diagnostic criteria have been reevaluated to include new associated features that were not initially considered and subclinical forms that were not originally diagnosed. Recent research has also shown that some genes responsible for syndromic CPO may also be candidate genes for non-syndromic CPO, further indicating that these conditions may represent different portions of a single spectrum (Stanier and Moore, 2004).

## Molecular biology of palatogenesis

Palatal development in mammals is a complex process involving a network of growth factors, cell-surface receptors, and signaling molecules. The palatal shelves appear around week 6 during gestation in humans (Chai and Maxson, 2006). They are composed of cranial neural crest-derived mesenchymal cells and mesoderm-derived endothelial cells, which together are covered by pharyngeal ectoderm-derived epithelial cells (Iwata et al., 2011; Hill et al., 2015). Normal development of the palate depends on proper migration, growth, differentiation, and apoptosis of these cells, and occurs in three major stages: vertical growth of the shelves down toward the sides of the tongue, elevation of the palatal shelves to acquire a horizontal position as the mandible lengthens, and fusion of the palatal shelves to form the transient midline epithelial seam, which ultimately undergoes epithelial to mesenchymal transition (Mossey et al., 2009; Sasaki et al., 2014). Cleft palate can arise due to an error in any of these stages, which many studies have demonstrated in mouse models. These defects can be grouped into five categories: failure of palatal shelf formation, fusion of the palatal shelf with the tongue or mandible, failure of palatal shelf elevation, failure of palatal shelves to meet post-elevation, and persistence of the medial edge epithelium (Chai and Maxson, 2006). However, it should be noted mouse models are more likely to demonstrate a cleft palate due to a smaller frontonasal prominence, making these studies limited in their applicability to humans, as gene mutations that cause CPO in mice may cause CL/P in humans.

Molecular heterogeneity along the anterior-posterior axis of the palate has been shown in mouse models, as evidenced by different gene expression, signaling pathways, and transcription factors between the anterior hard palate and posterior soft palate (Zhou et al., 2013). Additionally, specific gene mutations have been found to only cause clefts in the anterior or posterior palate. In the anterior palate, the associated genes are *Msx1, Bmp4*, and *Bmp2* in the bone morphogenetic pathways, *Shh* and *Spry2* in the sonic hedgehog (Shh) signaling pathway, *Fgf10* and *Fgf7* in the Fgf signaling pathway, the *Shox2* network, *Efnb1* in Ephrin signaling, and the Tgf-β family. The genes associated with the posterior palate are *Meox2, Tbx22*, and *Barx1*. Loss of function of *Shox2* results in an incomplete cleft of the anterior palate but normal posterior palate development, while mutations of *MSX1* in humans have been associated with isolated non-syndromic cleft palate (Smith et al., 2012). Many of these pathways are interrelated and mediate communication between the mesenchyme and the epithelium; for example, the BMP and FGF signaling pathways both utilize Shh signaling in the anterior palate to regulate palatal growth (Chai and Maxson, 2006). *Msx1* expression in the anterior mesenchyme regulates *Bmp4* expression, which regulates *Shh* expression in the epithelium. *Shh* in turn regulates *Bmp2* expression in the mesenchyme, and *Bmp2* expression regulates mesenchymal cell proliferation (Smith et al., 2012). FGF10 protein is expressed in the anterior palatal mesenchyme, and upon binding to its receptor FGFR2 in the palatal epithelium, induces *Shh* expression, forming a positive feedback loop (Chai and Maxson, 2006; Zhou et al., 2013). *Shh* functions to indirectly induce mesenchymal cell proliferation via *Bmp2*, and convergence of the BMP and FGF pathways on *Shh* expression in the anterior palatal epithelium controls anterior palatal outgrowth (Chai and Maxson, 2006).

## Descriptive epidemiology

Although the prevalence of CPO is unknown in some parts of the world, current reported rates for various countries are shown in Table 1. Prevalence of cleft palate varies greatly between certain regions. The highest reported rates are in British Columbia (a province of Canada), Finland, and Malta, with prevalence rates of 25.31, 14.31, and 14.13 per 10,000 live births, respectively. The lowest reported rates are in South Africa, Colombia, and Cuba, with prevalences of 1.93, 1.69, and 1.35 per 10,000 live births, respectively. In the U.S., the overall CPO prevalence is 5.9 per 10,000 lives births (Mai et al., 2014), and estimated to be 3.18 per 10,000 for non-syndromic CPO (Parker et al., 2010). Even though most countries calculate the prevalence of CPO from only live births, combined data from European registries for 1995–1399 reported 2.4% of babies with CPO were stillborn, and 8.1% were from terminated pregnancies (Mossey et al., 2009). It should be noted prevalence may be underestimated if using national birth registry lists, as cleft palates often go undiagnosed in utero or at birth due to their lack of external visibility (Cooper et al., 2006; Mossey et al., 2009).

**Table 1:** **Worldwide prevalence rates of cleft palate**.

**Country**	**Rate per 10,000 (95% CI if available)**	**References**
USA^*^	5.9 (5.7–6.0)	Mai et al., 2014
California	5.81 (5.54–5.98)	Saad et al., 2014
Kentucky	5.70	Human Genetics Programme, 2007
Alabama	5.37	Human Genetics Programme, 2007
Colorado	4.83	Human Genetics Programme, 2007
Hawaii	3.94	Human Genetics Programme, 2007
Rhode Island	2.22	Human Genetics Programme, 2007
Canada^*^	7.02 (6.58–7.47)	Mossey and Catilla, 2003
British Columbia	25.31	Mossey and Catilla, 2003
Alberta	8.07 (6.70–9.63)	Mossey and Catilla, 2003
**Caribbean**		
Cuba	1.35 (0.95–1.87)	Mossey and Catilla, 2003
**Central America**		
Mexico	3.06 (2.46–3.76)	Mossey and Catilla, 2003
Guatemala	4.7	Matute et al., 2014
**South America**		
Paraguay	6.83 (3.39–12.23)	Mossey and Catilla, 2003
Brazil	4.49 (3.65–5.47)	Mossey and Catilla, 2003
Colombia	1.69 (0.16–6.16)	Mossey and Catilla, 2003
**European Union**		
Finland	14.31(13.12–15.58)	Mossey and Catilla, 2003
Scotland	7.94 (5.95–10.39)	Mossey and Catilla, 2003
Malta	14.13 (10.13–19.16)	Mossey and Catilla, 2003
**Africa**		
South Africa	1.93 (1.49–2.46)	Mossey and Catilla, 2003
Nigeria	0.32	Butali et al., 2014a
**South Asia**		
India	1.7	Mossey and Little, 2009
Nepal	3.5	Singh et al., 2012
**East Asia**		
China	2.36 (1.98–2.78)	Mossey and Catilla, 2003
	2.6 (2.4–2.7)	Cooper et al., 2006
Japan	4.54 (4.02–5.10)	Mossey and Catilla, 2003
	3.7 (3.2–4.1)	Cooper et al., 2006
**Australia**	6.48	Mossey and Catilla, 2003
**New Zealand**	6.45	Mossey and Catilla, 2003

* *Top 3 highest and lowest rates per available states/provinces are provided*.

## Disparities in rates by sex and race/ethnicity

Cleft palate is more commonly reported in females than males, but the exact reasons why are still incompletely understood. The reported sex ratio of affected males to females by the World Health Organization is 0.93 (CI 95%: 0.89–0.96) for non-syndromic CPO (Mossey and Catilla, 2003). Female sex hormones may play a role in increased clefting for both lips and palates (Ross and Johnson, 1972; Miura et al., 1990), and as already noted, the female palate closes 1 week later than that of males. The suggested inheritance patterns for cleft palate are autosomal dominant and X-linked recessive. Although it may seem counterintuitive for an X-linked recessive condition to appear more often in females, X-chromosome inactivation in females in utero may explain the broader spectrum of phenotypes seen in females (Stanier and Moore, 2004; Jugessur et al., 2012). Although there is a 50% chance the mutated allele will inactivate in heterozygous females, leaving them affected less severely or not at all, females also have twice the risk of inheriting a mutated X-chromosome (Lidral et al., 2008).

Generally for all oral clefts, there are consistent patterns of increased risk in Asians whereas the lowest rates are seen in Africans (Mossey and Catilla, 2003). For CPO specifically, the highest rates were observed in whites and certain peoples from Canada and Northern European countries, while the lowest rates were again seen in Africans (Mossey and Catilla, 2003). Within the U.S., prevalence has been to shown to vary between different ethnicities, with the highest rates amongst non-Hispanic whites and American Indians or Alaska Natives and the lowest amongst non-Hispanic blacks. A national estimate with pooled data from 29 states reported the prevalence for isolated cleft palate as 6.4 per 10,000 live births for non-Hispanic whites, 6.4 for American Indians or Alaska Natives, 5.6 for Hispanics, 5.6 for Asians or Pacific Islanders, and 4.4 for non-Hispanic blacks (American Indian and Alaska Native groups may be skewed due to small sample size) (Mai et al., 2014). Within the state of California, different trends were shown, as prevalence rates were reported as 7.60 per 10,000 live births for non-Hispanic whites, 4.90 for Asian/Pacific Islanders, 4.79 for Hispanics, 4.12 for non-Hispanic blacks, and 2.11 for Native Americans (Saad et al., 2014).

The size of the facial processes may relate to cleft susceptibility, as size of the frontonasal process contributes to its ability to contact neighboring processes. Inherently smaller frontonasal processes, as found in Asians because of the combination of a smaller, flatter midface with a broader upper face, brachycephalic head, and elliptical palate, may contribute to their relative higher rates of clefting (Setó-Salvia and Stanier, 2014). Africans have broader, larger noses which imply a larger frontonasal process and larger palate; this may contribute to their lower clefting frequency (Eppley et al., 2005).

## Genetic risk factors

CPO has been shown to have a strong genetic component based on its high recurrence rate in families of affected individuals. In population studies, the relative risk of recurrence of CPO among first-degree relatives has been reported to be 56 times greater in Norway and 15 times greater in Denmark than the risk for the general population (Sivertsen et al., 2008; Grosen et al., 2010; Table 2). Twin studies have also shown monozygotic twins are more likely to both be affected by CPO than dizygotic twins. In Denmark, research has shown probandwise concordance rates of 33% for monozygotic twins and 7% for dizygotic. It was also noted that CPO and CL/P never both appeared in one pair of twins (Grosen et al., 2011).

**Table 2:** **Association between family history and risk of cleft palate**.

**Study**	**Population**	**Region**	**RR (95% CI)**
Sivertsen et al., 2008	Norway	All first-degree relatives	56 (37.2–84.8)
		Parents	54 (29.7–98.0)
		Siblings	58 (37.2–84.8)
Grosen et al., 2010	Denmark	All first-degree relatives	15 (13–17)
		Parents	10 (7–14)
		All Siblings	16 (13–20)
		Offspring	20 (15–26)

Currently, over 400 syndromes are reported to include CPO (Mossey et al., 2009). Syndromes including CPO that have been traced to a known gene mutation are listed in Table 3, along with their associated features. The most common form of syndromic oral clefts is Van der Woude's syndrome, which accounts for 2% of all CL/P cases and often presents with lower lip pits (Kondo et al., 2002; Brito et al., 2012; Leslie and Marazita, 2013). Most syndromes involving oral clefts present as either predominately CL/P or CPO, but Van der Woude's commonly shows both CL/P and CPO (Kondo et al., 2002). Mutations in *IRF6* cause Van der Woude's, along with popliteal pterygium (Leslie et al., 2015), and recent research suggests that mutations surrounding the gene may be involved in non-syndromic cleft palate (Zucchero et al., 2004; Rahimov et al., 2012; Pegelow et al., 2014). Another common form of syndromic clefting is DiGeorge (22q11.2 deletion) syndrome, which presents with heart and kidney abnormalities, hearing loss, and developmental delays (Burnside, 2015). DiGeorge patients may exhibit varying severities of cleft palate, including a type known as Pierre Robin Sequence (PRS), which presents with micrognathia and a malpositioned tongue, resulting in a distinctive U-shaped cleft palate (Brito et al., 2012). PRS is associated with mutations in a number of genes, including *SOX9* (Benko et al., 2009). Although PRS is considered non-syndromic when it presents alone, it is considered syndromic when it appears as a feature of other syndromes, such as DiGeorge syndrome, Stickler syndrome, or Campomelic dysplasia. These are among the major syndromes associated with palatal clefting; many forms of syndromic cleft palate have a prevalence of less than 1 out of 100,000, with only a handful of cases reported in the literature for some. Additionally, some conditions are caused primarily by *de novo* mutations and lack any family history, in contrast to non-syndromic cleft palate, which has a strong recurrence risk among affected families (Rahimov et al., 2012; Setó-Salvia and Stanier, 2014).

**Table 3:** **List of syndromes with a cleft palate and attributed genes**.

**Syndrome**	**Other associated features**	**Prevalence**	**Gene(s)**
Abruzzo-Erickson^†^	Coloboma, hypospadias, deafness, short stature, radial synostosis	4 cases reported^‡^	TBX22
Andersen^§^	Periodic paralysis, micrognathia, low-set ears, dental abnormalities, widely spaced eyes	100 cases reported	KCNJ2
Apert^*^	Craniosynostosis, syndactyly, sunken face, beaked nose, hearing loss	1/65,000–88,000	FGFR2
Bamforth-Lazarus^*^	Thyroid agenesis, choanal atresia	8 cases reported^‡^	FOXE1
CHARGE^*^	Coloboma, heart defect, choanal atresia, retarded growth and development, genital, and ear abnormalities (CP minor characteristic)	1/8500–10,000	CHD7
Cornelia de Lange^*^	Slow growth, intellectual disability, skeletal abnormalities, low-set ears, small and widely spaced teeth, small, and upturned nose	1/45,000–62,500^‡^	NIPBL, SMC1A, SMC3
Craniofrontonasal^§^	Hypertelorism, brachycephaly, downslanting palpebral fissures, clefting of nasal tip	Unknown	EFNB1
Crouzon^*^	Craniosynostosis, wide-set bulging eyes, shallow eye sockets, strabismus, beaked nose, underdeveloped upper jaw (CP minor characteristic)	0.9/100,000^‡^	FGFR2
Desmosterolosis^§^	Brain abnormalities, delayed speech and motor skills, muscle spasticity, arthrogryposis, short stature, micrognathia	10 cases reported	DHCR24
Diastrophic dysplasia^§^	Short stature, short arms and legs, early osteoarthritis, contractures, clubfoot, hitchhiker thumbs, swelling of external ears	1/100,000	SLC26A2
DiGeorge^*^	Heart abnormalities, breathing problems, kidney abnormalities, hearing loss, short stature, developmental delays	1/4000	TBX1, COMT
Hereditary lymphedema-distichiasis^§^	Limb lymphedema, distichiasis, astigmatism, varicose veins, ptosis, heart abnormalities	Unknown	FOXC2
Kabuki^§^	Arched eyebrows, long palpebral fissures w/everted lower lids, flat broadened nose, protruding earlobes, microcephaly, scoliosis, short fifth fingers, fetal finger pads	1/32,000	KMT2D, KDM6A
Kallmann—Type 1, Type 2^§^	Hypogonadotropic hypogonaidism, lack of secondary sex characteristics, anosmia, unilateral renal agenesis, hearing loss	1/10,000–86,000	KAL1 (Type 1), FGFR1 (Type 2)
Larsen syndrome; atelosteogenesis^§^	Clubfoot, hip/knee/elbow dislocations, extra bones in wrists/ankles, blunt and spatulate tips of fingers, scoliosis, frontal bossing, midface hypoplasia, wide-set eyes, hearing loss	1/100,000	FLNB
Lethal and Escobar multiple pterygium^*^	Pterygium, arthrogryposis, scoliosis, downslanting palpebral fissures, epicanthal folds, small jaw, low-set ears	Unknown	CHRNG
Loeys-Dietz, Types 1–4^*^	Craniosynostosis, scoliosis, pectus excavatum/carinatum, clubfoot, hypertelorism (bifid uvula and/or CP)	<1/1,000,000^‡^	TGFBR1, TGFBR2, SMAD3, TGFB2
Miller^*^	Malar hypoplasia, micrognathia, ectropion, lower eyelid coloboma, microtia (CP ± CL)	30 cases reported	DHODH
Oculofaciocardiodental^*^	Microphthalmia, broad nasal tip, atrial/ventricular septal defect, radiculomegaly	<1/1,000,000	BCOR
“Oro-facial-digital”^§^	Cleft tongue, broad flat nasal bridge, hypertelorism, syndactyly, (CP ± CL)	1/50,000–250,000	OFD1
Otopalatodigital Spectrum Disorders^*^:	Hearing loss from ossicle malformations, skeletal abnormalities, prominent brow ridges		FLNA
Type 1	Hypertelorism, downward-slanting eyes, small flat nose, Spatulate fingertips	<1/100,000	
Type 2	Hypertelorism, downward-slanting eyes, broad flat nose, micrognathia, camptodactyly	<1/100,000	
Frontometaphyseal dysplasia	Joint contractures, bowed limbs, scoliosis, hypertelorism, downward-slanting eyes, micrognathia	Few dozen cases reported	
Melnick-Needles	Short stature, scoliosis, partial dislocation of joints,bowed limbs, micrognathia, excess hair on forehead	<100 cases reported	
Pierre Robin Sequence^*^	Micrognathia, glossoptosis, failure to thrive	1/8500–14,000	SOX9
PRS w/Campomelic dysplasia	Bowing of leg bones, clubfoot, dislocated hips, ambiguous genitalia, small chin, prominent eyes, flat face, glossoptosis, micrognathia, laryngotracheomalacia	1/40,000–200,000	SOX9
PRS w/Stickler, Types 1–5	Flattened facial appearance, high myopia, abnormal vitreous, glaucoma, cataracts, retinal detachment, hearing loss, hypermobile joints, early-onset arthritis, scoliosis/kyphosis, platyspondyly	1/7500–9000	COL2A1, COL11A1, COL11A2, COL9A1, COL9A2
Popliteal pterygium^§^	Pits near center of lower lip, missing teeth, webs of skin on backs of knees, syndactyly, abnormal genitals (CP +/- CL)	1/300,000	IRF6
Saethre-Chotzen^*^	Craniosynostosis, ptosis, hypertelorism, broad nasal bridge, facial asymmetry, microtia	1/25,000–50,000	TWIST1
Smith-Lemli-Opitz^§^	Microcephaly, hypotonia, syndactyly, polydactyly	1/20,000–60,000	DHCR7
Snyder-Robinson^†^	Delayed development, hypotonia, scoliosis/kyphosis, prominent lower lip	10 cases reported	SMS
Treacher Collins^*^	Micrognathia, downward-slanting eyes, lower eyelid coloboma, microtia	1/50,000	TCOF1, POLR1C, POLR1D
Van der Woude^§^	Pits near center of lower lip, small mounds of tissue on lower lip (CP ± CL)	1/35,000–100,000	IRF6
X-linked cleft palate^*^	±Complete or partial ankyloglossia	Unknown	TBX22
X-linked intellectual disability:			
Siderius type^†^	Long face, sloping forehead, broad nasal bridge, upslanting palpebral fissures, low-set ears, large hands	Few cases reported	PHF8
Renpenning^†^	Developmental delay, short stature, upslanting palpebral fissures, shortened philtrum	60 cases reported	PQBP1

*All syndrome genes, associated features, and prevalences from Genetics Home Reference by the NIH unless otherwise specified (http://ghr.nlm.nih.gov/)*.

* *Syndromes listed in Leslie and Marazita (2013)*.

§ *Syndromes listed in Dixon et al. (2011)*.

† *Syndromes listed in Genetics Home Reference by the NIH*.

‡ *Prevalence from Orphanet (http://www.orpha.net)*.

Although many genome wide association studies (GWAS) and linkage studies have been done on non-syndromic oral clefts, most of them have focused on CL/P, identifying several genes with common variants. Studies that have compared non-syndromic CL/P with non-syndromic CPO found no association between CPO and the candidate genes for CL/P, providing further evidence that these two malformations have separate genetic etiologies (Böhmer et al., 2013). At the time of writing, there has been only one linkage study and two GWAS done solely on non-syndromic CPO, although many GWAS, linkage, and other association studies have been done on non-syndromic oral clefts as a whole, which include CPO. The results from some of these various studies are listed in Table 4.

**Table 4:** **Genetic risk factors for cleft palate**.

**Study**	**Country**	**SNP/nearby gene(s) (chromosome)**	**OR (95% CI) or *p*-value**
**LINKAGE STUDIES**			
Koillinen et al., 2005	Finland	1p34	*p* = 0.069
		2p24-p25	*p* = 0.016
		12q21	*p* < 0.05
**ASSOCIATION STUDIES**			
Pan et al., 2013	China	rs742071 (1p36)	0.85 (0.36–2.03)
		rs7590268 (2p21)	2.05 (1.07–3.91)
		rs7632427 (3p11.1)	1.00 (0.61–1.64)
		rs12543318 (8q21.3)	1.02 (0.68–1.52)
		rs8001641 (13q31.1)	1.52 (0.95–2.43)
		rs1873147 (15q22.2)	0.41 (0.20–0.86)
Butali et al., 2014b	Africa	c.493C > G (20q12)	Not listed
Nikopensius et al., 2010	Estonia	rs17389541 (1q32.3-q41)	1.726 (1.263–2.358)
	Latvia	rs1793949 (12q13.11)	1.659 (1.235–2.229)
	Lithuania	rs11653738 (17q21)	1.518 (1.123–2.053)
Carter et al., 2010	Ireland	rs3769817 (2q33.1)	1.45 (1.06–1.99)
		rs2166975 (2p13.3)	*p* = 0.041
Ghassibe-Sabbagh et al., 2011	Europe, USA Philippines	rs3827730 (1p32.3)	*p* = 0.0003

The sole linkage study focusing exclusively on non-syndromic CPO was performed using the DNA of 24 Finnish families, and involved scanning all of chromosomes 2 and 4 and a candidate region in 1p34 in both affected and unaffected family members, along with a genome-wide scan of nine of the families with larger pedigrees (Koillinen et al., 2005). Although no significant linkage was found for any gene, the results showed suggestive linkage for the loci 1p34, 2p24-p25, and 12q21, warranting further research on these areas as candidate regions for cleft palate. Although the study found no mutations in *IRF6*, the gene responsible for Van der Woude's syndrome, more recent research has shown that some genes responsible for syndromes involving cleft palate may also be candidate genes for non-syndromic cleft palate, with the most substantial evidence for the genes *IRF6* and *TBX22* (Marçano et al., 2004; Stanier and Moore, 2004; Zucchero et al., 2004; Nikopensius et al., 2010; Rahimov et al., 2012; Pegelow et al., 2014). In both of the GWAS on non-syndromic cleft palate, no single SNP was significant when considered alone (Beaty et al., 2011; Wu et al., 2014).

## Maternal and paternal risk factors

Although the risk factors for CL/P have been extensively researched through epidemiologic and experimental studies, few studies have focused exclusively on CPO. It is largely assumed that the risk factors for CPO are the same as for CL/P. These major risk factors include maternal exposure to tobacco smoke, alcohol, and corticosteroids; folic acid deficiency; zinc deficiency; and maternal grief. More recent research has differentiated between CPO and CL/P when analyzing their risk factors, and the odds ratios for CPO from some of these studies are listed in Table 5.

**Table 5:** **Maternal risks factors associated with cleft palate**.

**Risk factor**	**Study**	**Country**	**Categories**	**OR (95% CI)**
Smoking	Little et al., 2004	Various	Smoking vs. none	1.22 (1.1–1.35)
	Leite et al., 2014	Denmark	Smoking vs. none	1.09 (0.88–1.35)
	Butali et al., 2013a	Europe	Smoking vs. none	1.38 (1.04–1.83)
	Sabbagh et al., 2015	Various	Passive smoking exposure vs. none	2.11 (1.23–3.62)
Supplements	Butali et al., 2013a	Europe	Folic acid use vs. none	1.18 (0.89–1.57)
	Johnson and Little, 2008	Europe, North America, South America, Australia, Asia	Any supplement use vs. none	0.88 (0.76–1.01)
			Multivitamins vs. none	0.88 (0.74–1.04)
			Folic acid supplements vs. none	0.95 (0.79–1.14)
			Preconceptionally start vs. none	0.70 (0.51–0.98)
			After 4th month of gestation vs. none	0.99 (0.71–1.38)
Alcohol	Bell et al., 2014	USA, Australia, Europe, India, Brazil, Japan, Canada	Any alcohol use vs. no/low alcohol use	1.05 (0.92–1.21)
			Alcohol use during 1st trimester vs. no/low alcohol	1.05 (0.90–1.23)
			Alcohol use during pregnancy vs. no/low alcohol	1.06 (0.75–1.48)
			Binge drinking vs. no/low alcohol (1st trimester)	0.94 (0.74–1.21)
	Romitti et al., 2007	USA	1–4 drinks/mo vs. none	1.3 (1.0–1.9)
			5–15 drinks/mo vs. none	1.1 (0.8–1.7)
			16–30 drinks/mo vs. none	1.1 (0.6–1.8)
			>30 drinks/mo vs. none	1.1 (0.6–2.2)
Diabetes mellitus	Correa et al., 2008	USA	Pregestational DM vs. none	1.80 (0.67–4.87)
			Gestational DM vs. none	1.54 (1.01–2.37)
	Bánhidy et al., 2010	Hungary	DM Type 1 vs. none	2.2 (0.7–6.8)
			DM Type 2 vs. none	0.4 (0.1–3.2)
			Gestational DM vs. none	0.3 (0.0–2.0)
Obesity	Stott-Miller et al., 2010	Washington	Non-syndromic CP:	
			Overweight vs. normal weight	0.92 (0.69–1.22)
			Obese vs. normal weight	1.21 (0.85–1.72)
			All types of CP:	
			Overweight vs. normal weight	0.84 (0.66–1.08)
			Obese vs. normal weight	1.04 (0.76–1.42)
	Block et al., 2013	Florida	Pre-pregnancy BMI: underweight vs. normal	1.27 (0.91–1.77)
			Pre-pregnancy BMI: Overweight vs. normal	0.97 (0.79–1.20)
			Pre-pregnancy BMI: Obese vs. normal	1.32 (1.07–1.62)
	Stothard et al., 2009	Various	Obese vs. recommended BMI	1.23 (1.08–1.47)
			Overweight vs. recommended BMI	1.02 (0.86–1.20)
	Izedonmwen et al., 2015	Various	Obese vs. normal weight	1.14 (0.95–1.37)
			Overweight vs. normal weight	0.89 (0.75–1.06)
Nonsystemic corticosteroid use	Skuladottir et al., 2014	Norway	Syndromic CP:	
			All CST vs. none	1.68 (0.71–3.98)
			Dermatologic CST use vs. none	3.38 (0.87–13.09)
			Non-dermatologic CST use vs. none	1.08 (0.34–3.40)
			Non-syndromic CP:	
			Any type of CST use vs. none	1.30 (0.42–4.05)
			Dermatologic CST use vs. none	2.64 (0.49–14.31)
			Non-dermatologic CST use vs. none	0.83 (0.18–3.91)
Bereavement in antenatal period	Ingstrup et al., 2013	Denmark	Bereavement vs. none	1.34 (0.87–2.04)
			All types of bereavement vs. none	0.91 (0.45–1.82)
			Sudden death vs. none	1.69 (0.63–4.51)
			Death of a child vs. none	2.36 (1.09–4.92)
Environmental conditions	Chung et al., 2013	China (Hong Kong)	Sunshine at conception vs. none	*P* = 0.30
			Sunshine at 4 weeks vs. none	*P* = 0.072
			Sunshine at 8 weeks vs. none	*P* = 0.009
			NOx at conception vs. none	*P* = 0.506
			NOx at 4 weeks vs. none	*P* = 0.794
			NOx at 8 weeks vs. none	*P* = 0.343
			NO at conception vs. none	*P* = 0.127
			NO at 4 weeks vs. none	*P* = 0.795
			NO at 8 weeks vs. none	*P* = 0.085
Organic solvents	Desrosiers et al., 2012	USA	Chlorinated vs. none	0.83 (0.50–1.38)
			Stoddard vs. none	1.45 (0.72–2.87)
			Aromatic vs. none	1.03 (0.49–2.20)
Zinc (plasma levels)	Munger et al., 2009	Utah	Isolated CP:	
			9.3–10.4 vs. ≤ 9.2 μmol/L	0.75 (0.36–1.57)
			10.4–11.6 vs. ≤ 9.2 μmol/L	0.78 (0.39–1.54)
			≥11.7 vs. ≤ 9.2 μmol/L	0.93 (0.47–1.84)
			CP with Malformations:	
			9.3–10.4 vs. ≤ 9.2 μmol/L	1.03 (0.44–2.40)
			10.4–11.6 vs. ≤ 9.2 μmol/L	1.20 (0.55–2.65)
			≥11.7 vs. ≤ 9.2 μmol/L	0.67 (0.27–1.67)
	Tamura et al., 2005	Philippines	9.0–9.8 ≤ 8.9 μmol/L	0.65 (0.16–2.68)
			9.9–10.9 ≤ 8.9 μmol/L	0.27 (0.05–1.45)
			≥ 11.0 ≤ 8.9 μmol/L	0.07 (0.01–0.73)

Maternal exposure to tobacco smoke has been reported as the strongest risk factor for CPO. The most commonly reported odds ratio from one meta-analysis is 1.22, but a more recent study found an odds ratio of 1.38, with both studies comparing any tobacco smoke exposure against none at all (Little et al., 2004; Butali et al., 2013a). However, these values may be underestimated as most studies only assess active maternal tobacco use and not passive smoke exposure or paternal smoking (Mossey et al., 2009). This has been further evidenced by a meta-analysis on passive smoking which found an odds ratio of 2.11 for non-syndromic CPO (Sabbagh et al., 2015).

Although folic acid deficiency has been found to cause cleft palate in animal models, including a protective effect of folic acid supplements against trans-retinoic acid-induced cleft palate in mouse models (Yao et al., 2011), data on folate use and risk of cleft palate in humans have been inconclusive. Several clinical studies on maternal use of folate and risk of CPO showed no significant association between the two (Johnson and Little, 2008; Little et al., 2008; Li et al., 2012; Butali et al., 2013a), with one study reporting an odds ratio of 0.95 with folic acid use (Johnson and Little, 2008). However, these inconsistencies may partially be due to differences in methods of ascertainment for folic acid intake, as folic acid is available through dietary folate, folic acid supplements, or multivitamins with folic acid (Li et al., 2012). Some studies differentiate between these different forms while others do not, which may cause inconsistencies in the data if the dose of folic acid varies greatly between women. Additionally, even though there has been a reported decrease in oral clefts in North America since the mandatory fortification of grains with folic acid in the late 1990's (Parker et al., 2010; Saad et al., 2014), this decline is seen only in CL/P, while CPO rates have remained fairly constant (Johnson and Little, 2008).

Clinical studies on maternal alcohol consumption as a risk factor for CPO have been inconsistent as well (Meyer et al., 2003; Chevrier et al., 2005; Bille et al., 2007; Romitti et al., 2007), even though it has been shown in animal models to have a disruptive effect on neural crest cells, which contribute to lip and palate development (Bell et al., 2014). In one meta-analysis which stratified by the amount of alcohol consumption, the odds ratios only slightly varied, with 0.94 for binge drinking and 1.05 for any alcohol exposure (Bell et al., 2014). However, this seemingly low odds ratio and discrepancies between studies may be due to underreporting of alcohol use by mothers, as most people tend to underestimate how much they drink or overestimate the volume of “one drink.” More thorough studies still need to be done to establish maternal alcohol use as a risk factor for CPO, but obtaining accurate data may be difficult due to this recall bias.

Zinc is crucial to normal fetal development, especially in the central nervous system, and maternal deficiency has been shown to cause cleft palate in animal models (Hurley and Swenerton, 1966; Warkany and Petering, 1972; Quinn et al., 1990; Mossey et al., 2009). Although maternal zinc deficiency has been shown as a risk factor for oral clefts in human studies, data is still limited for CPO as most studies have analyzed CL/P (Krapels et al., 2004; Shah and Sachdev, 2006; Hozyasz et al., 2009); only two studies have examined CPO and maternal plasma zinc levels. In the Philippines, women with plasma zinc levels of 9.0–9.8 and 9.9–10.9 μmol/L had odds ratios of 0.65 and 0.27, respectively, and 94% of mothers of children with CPO were found to have low plasma zinc levels (defined as < 11.0 μmol/L) (Tamura et al., 2005). In Utah, for non-syndromic CPO, mothers with plasma zinc levels of 9.3–10.4 and 10.4–11.6 μmol/L had odds ratios of 0.75 and 0.78, respectively; for CPO with malformations, mothers with plasma zinc levels of 9.3–10.4 and 10.4–11.6 μmol/L had odds ratios of 1.03 and 1.20, respectively (Munger et al., 2009). When directly comparing the two studies, the odds ratios were not as significant in Utah as in the Philippines; however, maternal plasma zinc levels overall were higher in Utah, suggesting that maternal zinc deficiency may not be a strong risk factor unless it is severely compromised (Munger et al., 2009). Even though maternal zinc deficiency has been correlated with CPO in animal studies and with CL/P in humans, more clinical research is still needed to determine if low maternal zinc levels are a risk factor for CPO.

Other risk factors significant for CPO are corticosteroid use and bereavement in the antenatal period. In one study on oral nonsystemic corticosteroid use, syndromic CPO and any corticosteroid use had an odds ratio of 1.68, with an even higher odds ratio of 3.38 for dermatologic corticosteroids (Skuladottir et al., 2014). For non-syndromic CPO, all corticosteroid use had an odds ratio of 1.30, with dermatologic corticosteroids having an odds ratio of 2.64 (Skuladottir et al., 2014). For both syndromic and non-syndromic CPO, these odds ratios are more significant than those of smoking, folic acid deficiency, and maternal alcohol use. In a study on bereavement during the antenatal period (defined as the death of a close relative), an odds ratio of 1.34 was found for non-syndromic CPO; when male and female offspring were calculated separately, the odds ratios were 1.83 and 0.91, respectively; and when stress was due to the death of a child, the odds ratio was 2.36 (Ingstrup et al., 2013). This correlation of bereavement during the antenatal period and increased risk of oral clefts is thought to be due to the fact that stress causes increased levels of cortisol, which is a corticosteroid. Although more research is needed in this area, maternal exposure to corticosteroids of endogenous or iatrogenic origin alike has been shown to be a potential risk factor for CPO.

More recently, obesity and diabetes mellitus have been recognized as risk factors for cleft palate. Animal studies have shown that pregnant mice fed high-fat diets have a higher rate of offspring with cleft palates (Kappen, 2013). In a large case-control study of mothers who had pregestational (PGDM) or gestational diabetes mellitus (GDM) and children with birth defects, odds ratios of 1.80 and 1.54 were found for mothers of children with CPO who had PGDM or GDM, respectively (Correa et al., 2008). A similar study from Hungary found odds ratios of 2.2, 0.4, and 0.3 for mothers with DM Type 1, DM Type 2, and GDM, respectively (Bánhidy et al., 2010). Other research has also found an increased risk of CPO in children of women who had a pre-pregnancy body mass index of 30 or higher (Mandal et al., 2011; Block et al., 2013). A case-control study on mothers who were obese pre-pregnancy found an odds ratio of 1.21 for non-syndromic CPO and 1.04 for all forms of CPO (Stott-Miller et al., 2010). A systematic review and meta-analysis by Stothard et al. found an odds ratios of 1.23 for pre-pregnancy obesity and all forms of CPO, and a similar study by Izedonmwen et al. found an odds ratio of 1.14 (Stothard et al., 2009; Izedonmwen et al., 2015). Although the mechanism by which obesity may contribute to cleft palate is still unknown, it is hypothesized it may be due to nutritional deficiencies or to alterations in glycemic control, similar to those experienced by diabetic mothers (Izedonmwen et al., 2015). One study has shown changes in lipid metabolism in mice with aberrant TGF-β signaling contribute to cleft palate formation, indicating a potential mechanistic link between diabetes mellitus and cleft palate (Iwata et al., 2014). Even though clinical studies have been fairly consistent in their findings for diabetes mellitus and obesity as risk factors for CPO, more research is still needed on the pathophysiology of this process.

Paternal risk factors for CPO have not been thoroughly researched, with the majority of studies focusing on advanced paternal age (Green et al., 2010; Bell et al., 2014; Ma et al., 2015). In a large study on congenital malformations in Poland, an odds ratio of 1.11 was found with increasing paternal age every 5 years (Materna-Kiryluk et al., 2009), and a meta-analysis on parental age and oral clefts found a 58% higher probability of CPO in children of fathers 40 years and older (Herkrath et al., 2012). Krapels et al. performed one of the largest questionnaire studies to date on several paternal risk factors, finding odd ratios of 1.5 for smoking, 1.8 for alcohol use, 0.6 for coffee use, 0.5 for medication use, and 0.2 for allergies (Krapels et al., 2006). Besides older paternal age, it is still unclear if paternal factors increase the risk of cleft palate formation.

## Gene-environment (GxE) interactions

Several study designs have been implemented to examine the possible role of interactions of environmental teratogens with genetic mutations on cleft palate formation. Skare et al. analyzed case-parent trios against control-parent trios, using known candidate genes for cleft palate, and found a potential interaction between *TBX4* (chromosome 17q21-q22) and dietary folate (Skare et al., 2012). A meta-analysis was performed by Zeiger et al. on oral clefts and maternal smoking, which found an odds ratio of 1.95 for the transforming growth factor alpha (*TGFA*) TaqI C2 allele and CPO (Zeiger et al., 2005).

To date, only two GWAS have analyzed gene mutations and environmental interactions for non-syndromic CPO. Beaty et al. analyzed a gene's risk based on maternal exposure to three common environmental risk factors, using an international consortium of 550 case-parent trios (Beaty et al., 2011). Although no single SNP was significant when considered without maternal exposure, certain SNPs in several loci showed a strong association with CPO when GxE interactions with maternal smoking, alcohol use, and vitamin use were included. For maternal smoking, several SNPs reached genome-wide significance in the genes *MLLT3* and *SMC2*, both on chromosome 9, and *OBSCN* on chromosome 1q42.13. For maternal alcohol use, SNPs were found to reach genome-wide significance in the genes *TBK1* on chromosome 12q14.2 and *ZNF236* on chromosome 18q22-q23. SNPs in genes *BAALC* on chromosome 8q22.3 and *ACOXL* on chromosome 2q13 were found to have a greater protective effect with maternal vitamin use. Wu et al. performed a similar GWAS stratifying trios into Asian and European ancestry (Wu et al., 2014). Several SNPs in the genes *SLC2A9* and *WDR1*, both on chromosome 4p16.1, in the Asian trios approached genome-wide significance when maternal environmental tobacco smoke exposure was considered. The most significant SNPs included rs3733585 and rs12508991 in *SLC2A9* (*p* = 2.26 × 10^−7^ and 2.26 × 10^−7^) and s6820756 and rs7699512 in *WDR1* (*p* = 1.79 × 10^−7^ and 1.98 × 10^−7^, respectively). Although chromosome 4p16.1 has been implicated as an additional contributor to non-syndromic CL/P, the candidate genes found on chromosomes 1, 2, 8, 9, 12, and 18 are all distinct from those found in GWAS of non-syndromic CL/P (Beaty et al., 2010; Butali et al., 2013b), further suggesting that CL/P and CPO are separate malformations.

## Morbidity and mortality

Children born with oral clefts have been shown to have higher mortality rates, especially in the presence of other birth defects (Vallino-Napoli et al., 2006; Carlson et al., 2013). Kang et al. found a 15 times greater risk of mortality in CPO patients when compared to the general population, and a 10 times greater risk when compared to other types of clefts (Kang et al., 2012). A 14-year study of Dutch patients found an infant mortality rate (IMR) of 2.45% for all CPO, with the most common cause of death for all oral clefts being congenital malformations of the heart (40.6%; van Nunen et al., 2014). Congenital heart defects commonly present with oral clefts, and are reported to occur in 1.3 to 27% of affected individuals, although the mechanism is still unknown (Setó-Salvia and Stanier, 2014).

Epidemiological studies have assessed the relationship between cancer and clefts. Bille et al. found an increased risk of breast cancer and primary brain cancer in females with cleft palate (Bille et al., 2005), while Lima et al. found breast, colorectal, stomach, prostate, and uterine cancers to be the most common among those with oral clefts (Lima et al., 2013). Recent research has also shown differences in cerebellar morphology in patients with oral clefts. DeVolder et al. found that males with cleft palate had regional changes in the cerebellum but not reductions in volume, while females with cleft palate had reduced cerebellum volumes (DeVolder et al., 2013).

## Treatment

Multidisciplinary care is needed to provide comprehensive treatment for CPO beginning at birth and spanning until adulthood. Care for children born with these defects includes plastic surgery, nursing, maxillofacial surgery, otolaryngology, speech therapy, audiology, psychological counseling, genetic testing and counseling, dentistry, and orthodontics. While each cleft center has developed its own team approach and sequence of care, typical management involves the following, described here briefly.

### Surgical treatment and complications

Unlike the artistic nature of the cleft lip repair, the cleft palate repair is very functional in nature. The goal of the surgery certainly includes closure of the defect, but mostly focuses on quality of speech (Agrawal, 2009). Multiple different methods of repair have been demonstrated and improved throughout the years, focusing on either lengthening of the palate, alignment of the muscle or both (Strong and Buckmiller, 2001). Soft palate repair techniques may be used in isolation or combined with hard palate procedures, as necessary. Most surgeons today perform either some modification of an intravelar veloplasty, vs. a two-flap palatoplasty with double opposing z-plasty to achieve levator muscular repositioning (Sitzman and Marcus, 2014). Overall, the goals of palate repair are separating the oral and nasal cavity and creating a competent velopharyngeal valve for swallowing and speech, while preserving midface growth and development of functional occlusion (Friedman et al., 2010).

The timing of repair is also debated, and has ranged from shortly after birth to as late as 6 years of life. Much of the controversy against the early repair centers on inhibition of facial growth, in contrast to the late repair, which is met with significant restrictions in clear speech. Today, most cleft surgeons focus on the type of repair to be performed in a period somewhere between 9 and 18 months of age. The palate repair technique and timing chosen by each surgeon is heavily reliant on their training, comfort and preference given the lack of long-term evidence of efficacy and outcomes. A 2007 survey of 306 American cleft surgeons showed that 96% perform one-stage repairs and 85% perform palate surgery when the patient is between 6 and 12 months of age. Evidence suggests that children do not benefit from palate repair after age seven, as significant speech abilities have already developed and changing the anatomy at this stage may hinder speech progress. Early interventions such as nasoalveolar molding, presurgical orthopedics, external taping, and gingivoperiosteoplasty are newer advances that aim to minimize the number of surgeries needed and optimize surgical results by repositioning bony and soft tissue structures prior to an infant's first palate surgery (Hopper et al., 2006).

Immediate complications of cleft palate repair are bleeding, respiratory obstruction, infection, and dehiscence. Bleeding and respiratory obstruction happen immediately after surgery, and while rare require re-intubation and may be life-threatening (Hopper et al., 2006). Palatal (oronasal) fistulas may also form, ranging from asymptomatic holes to large communications between the oral and nasal cavities that cause speech problems, nasal regurgitation and hygiene difficulties. If symptomatic, fistulas may be surgically corrected with local mucosal flaps (Katzel et al., 2009). Factors that affect fistula formation include the anatomy of the cleft (primary palate clefts have higher fistula rates), the type of repair, and the experience level of the surgeon (Hopper et al., 2006).

### Long-term treatment

Even though repairing the cleft palate itself may be a one-time operation, treating the resulting dental and speech problems, along with the associated psychological implications, is a long-term effort usually not fully completed until the late teenage years (Setó-Salvia and Stanier, 2014). Much of the debate regarding long-term outcomes of cleft repairs is centered on speech development and growth of the mid-face. Inadequate repair of the palatal muscles or inadequate length of the soft palate after palatoplasty may result in a structural defect or physiologic dysfunction of the velopharyngeal valve, resulting in the most common speech deficiency after cleft palate repair: velopharyngeal insufficiency (VPI). The inability to completely separate the oral and nasal cavities during speech leads to hypernasality, nasal emission, imprecise consonant pronunciation, decreased vocal loudness, and speaking in short phrases (Hopper et al., 2006). In a study on the health-related quality of life (HRQL) of children with oral clefts, researchers found that HRQL decreased as severity of speech problems increased, and older children with CPO had lower HRQL than those with CL/P (Damiano et al., 2007). Monitoring for early VPI can be done through speech therapy and nasopharyngoscopy both before and after palate surgery (Chen and Kane, 2012). Surgical options for improving VPI are a pharyngeal flap or sphincter pharyngoplasty.

Decreased maxillary width and crossbite may be treated with orthodontic expansion combined with bone grafting. Midface hypoplasia (restricted growth of the mid-face) often results in an Angle Class III occlusion (underbite) and may be treated with a distraction device and eventual surgery to advance the midface (LeFort I maxillary orthognathic advancement) (Hopper et al., 2006).

## Future directions for research

Five main themes emerge as critical in advancing research in CPO: (1) monitoring of the occurrence of CPO across different parts of world to assess prevalence and availability of health care services (capacity building); (2) detailed phenotyping of the severity of CPO in relation to timing in embryogenesis and potential genetic/environmental factors that can impair closure (biology); (3) understanding of the genetic and environmental risk factors for CPO and their interaction (primary prevention); (4) access to early detection and multidisciplinary treatment of children with CPO from birth into adulthood (clinical services); and (5) understanding predictors of recurrence and possible interventions to lower risk among mothers of reproductive age (secondary prevention). In all five areas, it will be important to distinguish between CPO and CL/P in order to address potential differences, which will present challenges given the rarity and less obvious nature of CPO. This will necessitate large consortium efforts globally to attain a large enough sample size to evaluate CPO-specific genetic and environmental risk factors, multidisciplinary approaches to treatment and predictors of future recurrence in parents with at least one affected child.

## Author contributions

MB acquired references and data and contributed to paper design, drafting, revising and final approval of the manuscript, ensuring accuracy and integrity of the paper. YC and WM contributed to paper design and critical revisions and final approval of the manuscript, ensuring accuracy and integrity of the paper. CY acquired references and data and contributed to drafting and final approval of the manuscript, ensuring accuracy and integrity of the paper. JF conceived paper idea and design and contributed to drafting, revising and final approval of the manuscript, ensuring accuracy and integrity of the paper.

### Conflict of interest statement

The authors declare that the research was conducted in the absence of any commercial or financial relationships that could be construed as a potential conflict of interest.
